# Localization of Low Copy Number Plasmid pRC4 in Replicating Rod and Non-Replicating Cocci Cells of *Rhodococcus erythropolis* PR4

**DOI:** 10.1371/journal.pone.0166491

**Published:** 2016-12-09

**Authors:** Divya Singhi, Aayushi Jain, Preeti Srivastava

**Affiliations:** Department of Biochemical Engineering and Biotechnology, Indian Institute of Technology, Delhi, India; Florida International University, UNITED STATES

## Abstract

*Rhodococcus* are gram-positive bacteria, which can exist in two different shapes rod and cocci. A number of studies have been done in the past on replication and stability of small plasmids in this bacterium; however, there are no reports on spatial localization and segregation of these plasmids. In the present study, a low copy number plasmid pDS3 containing pRC4 replicon was visualized in growing cells of *Rhodococcus erythropolis* PR4 (NBRC100887) using P1 *parS*-ParB-GFP system. Cells were initially cocci and then became rod shaped in exponential phase. Cocci cells were found to be non-replicating as evident by the presence of single fluorescence focus corresponding to the plasmid and diffuse fluorescence of DnaB-GFP. Rod shaped cells contained plasmid either present as one fluorescent focus observed at the cell center or two foci localized at quarter positions. The results suggest that the plasmid is replicated at the cell center and then it goes to quarter position. In order to observe the localization of plasmid with respect to nucleoid, plasmid segregation was also studied in filaments where it was found to be replicated at the cell center in a nucleoid free region. To the best of our knowledge, this is the first report on segregation of small plasmids in *R*. *erythropolis*.

## Importance

Plasmid replication and segregation are fundamental processes required for maintenance. In the present study, we report the localization of a low copy number plasmid pRC4 in *Rhodococcus erythropolis* PR4. We demonstrate that plasmid replication takes place only when the cells begin to change their shape from cocci to rod. The findings were validated by localization of replisome, which shows that the cocci cells were non-replicating. The results suggest that the cells were in a quiescent stage when they were cocci. The replication machinery is turned on only when the shape changes to rod. Thus, cytoskeletal structures may have a role in plasmid segregation. Further, the tools developed in the present study can be used for tracking chromosomal loci in future.

## Introduction

*Rhodococcus* are versatile and are known to have the ability to degrade a large variety of compounds [1]. To exploit the diverse metabolic potential, it is very important to have the basic understanding of cell physiology and other vital processes such as replication and segregation. The name *Rhodococcus* originates from the fact that the bacterium can exist in different shapes, rod and cocci. The bacteria can also form filaments with short projections. These filaments undergo fragmentation to give rise to rod or cocci shaped cells [2]. This feature makes it an interesting bacterium for plasmid segregation studies as one can study the localization and segregation in different morphologies of the same cell.

Plasmid segregation studies have been largely done on plasmids originating from *E*. *coli* such as ColE1, P1, F, RK2 etc. In case of P1, F and RK2, plasmid was found to be replicated at the cell center and rapidly goes to quarter position [3]. ColE1 on the other hand, was localized at the cell poles [4]. Most low copy number plasmids utilize active partitioning systems. There are four types of partitioning systems reported viz. Type I, II, III and IV. Type I reported in case of F plasmid as *sopABC* encodes for a P loop ATPase that oscillates across the nucleoid and the plasmids are pulled to the quarter position by filament disassembly [5]. Type II observed in case of R1 plasmid as *parRMC* encodes for an actin like ATPase that forms filaments and pushes sister plasmids to the poles [5]. Type III system uses tubulin like GTPases reported in case of pBtoxis and Type IV observed in plasmid pSK1 uses a single non-NTPase coiled coil protein [6]. In case of plasmid R388, segregation without active partition has been demonstrated. R388 does not carry Par proteins and plasmid segregation follows a pilot fish mechanism where segregation is ensured by host chromosome segregation machinery [7]. In low copy number small plasmids such as in pSC101, pLS11 and pYAN-1 a cis sequence has been reported which does not encode for any protein but stabilizes unstable plasmids [8–10]. High copy number plasmids such as ColE1, on the other hand are randomly segregated and do not require a partitioning apparatus [11].

Only limited studies on plasmid localization and segregation have been done on plasmids originating from Actinomycetes family. To the best of our knowledge no such studies have been done in *Rhodococcus*.

A number of plasmids have been reported from different species of *Rhodococcus* [12]. The size of the plasmid varies from 5 kb to 510 kb [12]. A small low copy number plasmid pRC4 (from *Rhodococcus rhodochrous*) that can replicate in *Rhodococcus erythropolis* PR4 was selected for segregation studies.

In Rhodococci the growth cycle begins with cocci, which grow into rods and then filaments. These filaments undergo fragmentation to give rise to cocci and short rods. The stage at which plasmid replication and/or segregation take place is not known. There are three possibilities. 1) Plasmid replication begins while the cells are cocci and plasmid segregation takes place when the cells become rod shaped; 2) Plasmid replication and segregation takes place in cocci cells; 3) Plasmid replication begins only when the cells become rod shaped or during their transition to rod shape which is further followed by segregation. The question thus arises as to which model is being followed in *Rhodococcus*.

## Materials and Methods

### Bacterial Strains, plasmids and growth conditions

Bacterial strains and plasmids used in the present study are listed in Table 1. *E*. *coli*, *R*. *erythropolis* PR4 strains were cultivated in Luria broth at 37°C and 30°C respectively. Antibiotics kanamycin (50 μg/ml), ampicillin (100 μg/ml) and chloramphenicol (25 μg/ml) were used wherever necessary.

**Table 1:** Bacterial strains and plasmids used in the present study.

Bacterial strains and plasmids	Relevant description	References
*Escherichia coli* DH5α	Strain for cloning	Invitrogen
*Rhodococcus erythropolis* PR4	Sequenced strain of *Rhodococcus*	NBRC100887
*Escherichia coli* dnaBts		Kind gift of Dr. Dhruba Chattoraj, NCI, NIH
**PLASMIDS**		
pRSG43	*E*. *coli*-*Rhodococcus* shuttle vector, 5241 bp, Kan^R^	[26]
pEPR1	Shuttle promoter probe vector replicating in *E*. *coli* and PR4,7345 bp, Kan^R^	[27]
pDS132	Suicide vector for conjugal transfer and integration,5286 bp, Cm^R^	[28]
pPS68	Supplies GFP-ParB,5826 bp, Amp^R^	[19]
pPS89	Supplies *par*S-Kan,4398 bp, Amp^R^ and Kan^R^	[19]
pDS1 (pEPRI-Cm^R^)	8087 bp,Cm^R^	this study
pDS3 (pRSG43+pPS89)	8030 bp; Kan^R^	this study
pDS2 (pEPRI-Cm + pPS68)	9895 bp; Cm^R^	this study
pDS4	7657bps; Kan^R^	this study
pAJ01	7371bps; Kan^R^	this study
pAJ02	9093bps; Kan^R^	this study

Plasmids were isolated using QIAprep Spin Miniprep Kit (QIAGEN, Germany) following manufacturer’s instructions. Plasmid isolation, restriction enzyme digestion, ligation, electroporation and transformation were performed using standard methods as mentioned in Sambrook et al [13]. Restriction enzymes and ligase were obtained from NEB, USA.

### Construction of plasmid pDS1 (pEPR1+Cm)

A two-plasmid system was developed based on the replicons pCG1 and pRC4. Plasmids pEPR1 and pRSG43 were used as the source of pCG1 and pRC4 replicon respectively. The P1 parS was cloned in plasmid pRSG43 and P1 ParBGFP in another plasmid pEPR1. Since, both the plasmids pRSG43 and pEPR1 contained kanamycin resistance gene marker, the marker was replaced from one of the plasmid. Thus a chloramphenicol cassette was introduced in the vector pEPR1, which disrupted the kanamycin resistance gene marker.

For, this purpose, plasmid pEPR1 was partially digested with FspI and the larger 7.3 kb linearized plasmid was ligated with chloramphenicol resistance gene cassette (CmR cassette) of size approximately 740 bp obtained by the digestion of pDS132 with restriction enzymes EcoRV and Sma I. The colonies obtained after transformation and plating on LA plates containing chloramphenicol were further confirmed by plasmid isolation and restriction digestion. The plasmid was named as pDS1.

### Construction of plasmid pDS2 (pDS1+ pPS68)

Plasmid pDS1 constructed above was digested with restriction enzymes Pst I and Sma I and the large fragment of approximately 6.7 kb was ligated with 3.1 kb fragment of interest containing P1 ParBGFP cassette obtained from pPS68 by digesting with restriction enzymes Nsi I and Sma I. Colonies obtained after transformation and plating on LA plates containing chloramphenicol and kanamycin were further confirmed by plasmid isolation and restriction digestion. The plasmid constructed namely, pDS2 was used as the source of ParB-GFP.

### Construction of plasmid pDS3 (pRSG43+pPS89)

Plasmid pRSG43 was digested with restriction enzymes Fsp I and Pst I, and fragment of size 5060 bp containing pRC4 replicon was gel eluted. It was ligated with 2.9 kb fragment containing *parS-kan* cassette obtained from pPS89 after digestion with restriction enzymes Pst I and then with Sma I. The colonies obtained after transformation and plating on LA plates containing kanamycin were further confirmed by plasmid isolation and restriction digestion. The newly constructed plasmid containing pRC4 replicon was named as pDS3 and was used for studying segregation.

### Construction of plasmid expressing DnaB-GFP

The gene encoding for green fluorescence protein (GFP) 714 bp, was amplified using plasmid pALA2705 as template and primer pairs (BGFPF TCGATCTAGAGGCCAGCTGATGAGTAAAGGAGAAGAACTTTTCA and XGFPR GCATGTCGACGAGCTCGAATTCTTTGTATAGTTCATCCATGCC). The amplicon digested with restriction enzymes XbaI and SalI, was ligated with 6648 bp fragment obtained from pEPR1 by digestion with restriction enzymes XbaI and SalI. Colonies obtained after transformation of the ligated product were confirmed by plasmid isolation and restriction digestion with XbaI and SalI for the release of insert. The plasmid constructed was named pAJ01. The gene encoding for DnaB alongwith its native promoter was amplified using genomic DNA of *R*. *erythropolis* PR4 and primer pairs (DnaBF TCGAATGCATTATGCGGACCTACTAGTCCCTAACGTCATC and DnaBR TCGATCTAGATCCCCGGGCCATGTCG). The PCR product was digested with restriction enzymes NsiI and XbaI and ligated with pAJ01 digested with NsiI and XbaI. Colonies obtained after transformation were screened by plasmid isolation and restriction digestion. The plasmid constructed was named pAJ02. The *dnaB-GFP* cassette was also cloned in pRC4 replicon containing plasmid. For this purpose, the fragment of size 5060 bps obtained from pRSG43 digested with restriction enzymes FspI and PstI was ligated with 2768 bps fragment containing *dnaB*-*gfp* obtained from pAJ02 after digestion with restriction enzymes NsiI and EcoRV. The colonies obtained after transformation of the ligated product on LA kanamycin plates were confirmed by colony PCR with primers of *dnaB* and by plasmid isolation and restriction digestion. The plasmid constructed was named pDS4.

### Fluorescence microscopy

Exponentially growing cells in LB medium with OD_600_ ~ 0.2–0.3 were concentrated by centrifugation at 2,500 rpm for 5 min. and washed twice with 50 mM HEPES Buffer (pH 7.4) (Fisher Scientific, Waltham, MA). Approximately 2.5 μl of cells was placed on a slide and overlaid with a coverslip treated with poly-L-lysine (Sigma-Aldrich, St. Louis, MO) before microscopy. For localization studies, foci were visualized with Nikon motorized upright research microscope, Ni-E model. DS-Qi2 monochrome microscope camera was used to acquire and capture fluorescence signals with assistance of software NIS-elements BR and foci were measured using VImage public domain software.

### Plasmid segregational stability studies

Stability and compatibility assay of two plasmid system was carried out as described earlier by Srivastava et al [14] by dilution plating on antibiotic free LA plates and the colonies obtained were replica plated on chloramphenicol and kanamycin antibiotic containing plates.

### Nucleoid staining

The staining of nucleoids was done by incubation with DAPI (4’,6’-diamidino-2-phenylindole) at 50 μg/ml for 10 min. at room temperature before sampling the cells on the microscope slide.

### Flow cytometry

The samples for flow cytometry were prepared as described by Srivastava et al., 2006 [15]. Briefly, *R*. *erythropolis* PR4 electroporated with the plasmid pDS4 or pAJ02 were inoculated in 5 ml LB and incubated at 30°C, 200 rpm till the OD_600_ reached 0.3–0.5. The cultures were then treated with rifampicin (150 μg/ml) and cephalexin (10 μg/ml), which stop RNA synthesis and cell division respectively. The cultures were further incubated for 5 h. The cultures were chilled and 1.8 ml culture of these was pellet down at 6000 rpm for 5 min. The pellets were washed twice with 1 ml 1x PBS containing 1 mM EDTA (PBSE). The pellet thus obtained was resuspended in 100 μl of PBSE and 900 μl of 77% ethanol. The fixed cell samples were stored at—20°C.

Before the cells were to be analyzed by flow cytometry, the fixed cell samples were washed twice and resuspended in PBSE buffer. OD_600_ was noted and an aliquot was made such that it contained 10^7^ cells. The volume of 900 μl was made up by PBSE. To this, 100 μl of DAPI was added such that the final concentration became 50 μg/ml. It was left to be stained for 10 min at room temperature.

The samples were injected in BD Calibur. The software used was FACSDiva version 6.1.3.

## Results

The studies on spatial localization of small plasmids have been mostly limited to *E*. *coli* and *Bacillus subtilis*. In the present study we demonstrate the spatial localization of a small low copy number plasmid, pRC4 in *R*. *erythropolis* PR4. We show that cocci cells are non-replicating and plasmid replication and segregation takes place only in rod shaped cells. Further, plasmids were localized in nucleoid free region.

### Strategy for Localization of plasmid pRC4

For localization of pRC4 replicon, the P1 parS-ParB-GFP system developed by Dr. Stuart Austin and coworkers was used [16]. The system consists of a plasmid, which has ParB from plasmid P1 translationally fused to GFP under the control of an IPTG inducible *lac* promoter. The P1 parS is integrated in the chromosome/plasmid and the loci are tracked because of the binding of ParB-GFP specifically to P1 parS. The system was initially developed for tracking chromosomal and plasmid loci in *E*. *coli* [17,18]. Later it was used for tracking chromosomal loci in other bacteria such as *Vibrio cholerae* [19] etc.

In the present study, the P1parS-ParB-GFP system has been used for tracking a small plasmid in *R*. *erythropolis* PR4. Plasmid pDS3 containing pRC4 replicon was used for localization. The P1 parSkan cassette obtained from plasmid pPS89 was cloned into pRSG43 at a non-essential region away from the replication and maintenance genes to get the plasmid pDS3. For visualization of the plasmid, P1 ParB-GFP was supplied in trans from plasmid pDS2, which contained pCG1 replicon. The strategy is that the plasmid containing P1 parS cassette can be tracked in transformants containing another plasmid supplying P1 ParB-GFP.

### Stability and compatibility studies of plasmids pDS2 and pDS3

However, such a two-plasmid system would be functional only if the two plasmids are stable and compatible. Stability of individual plasmids was determined in *R*. *erythropolis*. The plasmid pDS3 was found to be 100% stable in *R*. *erythropolis* PR4 up to 60 generations without selection pressure (S1A Fig). The cloning of parS cassette from P1 did not perturb the structural or segregational stability of the plasmid. On the other hand, plasmid pDS2 containing pCG1 replicon (from *Corynebacterium glutamicum*) was found to be segregationally less stable. Only 60% colonies were found to contain plasmid as observed by replica plating and plasmid isolation. Similar results were obtained when plasmid pEPR1 (pCG1 replicon) without ParB-GFP was used (S1B Fig).

When the two plasmids were electroporated in *R*. *erythropolis* and grown in the absence of selection pressure (chloramphenicol and kanamycin), plasmid pDS3 was stably maintained and plasmid pDS2 was found to be segregationally less stable. On replica plating on plates containing either, kanamycin or chloramphenicol alone or in the presence of both the drugs, it was found that 50% cells did not grow on chloramphenicol plates which was used for selecting the plasmid pDS2 and double antibiotic plates (S1C Fig). On the other hand, 100% growth was observed in case of kanamycin plates used for selecting for the plasmid pDS3. The plasmids were isolated from several colonies picked from antibiotic containing plates and agarose gel electrophoresis was performed. Both the plasmids were found to be structurally stable (data not shown).

When the same experiment was performed in the presence of antibiotics, all the cells were found to contain both the plasmids. The results suggest that plasmid pCG1 and pRC4 can be used to develop a two-plasmid system only in the presence of selection pressure. In the absence of selection pressure, there is selective loss of one of the plasmid.

### Spatial Localization of plasmid pDS3 (pRC4 replicon)

Since the plasmids were stably maintained in the presence of selection pressure, we used them to develop a two-plasmid system. The two plasmids, pDS2 and pDS3 were electroporated in *R*. *erythropolis* PR4 and transformants were selected on chloramphenicol and kanamycin plates. Single colonies were selected and grown in LB media containing respective antibiotics. The culture was induced with 0.1 mM IPTG at an OD_600_ ~0.2. Exponentially growing cells were used for visualization under the microscope. Morphologically, the cells were cocci, short rods or long rods. Very little or no branching was observed.

**Fig 1:**
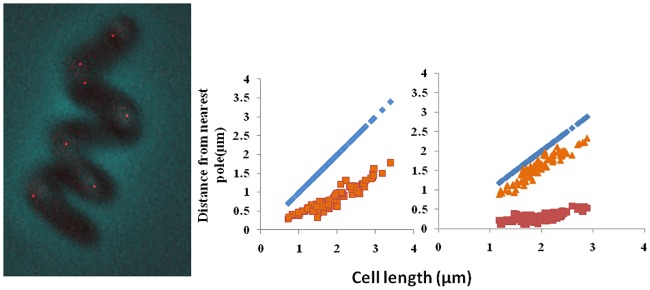
Intracellular Localization of plasmid pDS3 in growing cells of *R*. *erythropolis* PR4. a) Fluorescence microscopy images demonstrating the subcellular positioning of plasmids showing one focus or two foci; b) Subcellular distribution of plasmid pDS3 in *R*. *erythropolis* PR4 grown in LB medium at 30°C. Graphs show data of cells containing one focus or two foci. Cell length (in microns) is shown against distance from one pole.

When visualized under microscope, cells with either one or two fluorescence foci were observed (Fig 1A). No cells were observed with 3 or more fluorescence foci were observed. The number of one and two fluorescence foci cells out of the total number of cells showing fluorescence was about 47 and 53% respectively. Fluorescence was observed in about 70–80% cells. In cells containing only plasmid pDS2, diffuse fluorescence throughout the cell was observed and no discrete foci were observed in any of the cells.

In small cells, plasmid pDS3 was found to be localized in the center. Cells containing one focus ranged in size from 0.7 μm to 3.3 μm. Cells with two foci were also observed. The cells with two foci varied in length from 1.5 μm–3 μm. The fluorescent foci were localized at the one fourth and three fourth (quarter) positions (Fig 1B).

The newly born cells had one fluorescence focus in the center suggesting that the plasmid might be replicated at the center.

### Spatial Localization of plasmid pRC4 in cocci cells

**Fig 2:**
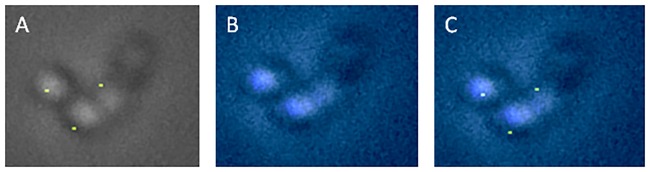
Localization of plasmid pRC4 in cocci cells. Representative images of cocci cells carrying plasmid pRC4 visualized using parS-ParB-GFP system. A) GFP fluorescence showing plasmid pRC4 B) nucleoid stained by DAPI and C) overlay of both plasmid and nucleoid.

To follow the localization of plasmid pRSG43 in cocci cells, culture in early lag phase was used. There was a mix of cocci cells and short rods. It was found that cocci cells were having one fluorescence focus only (Fig 2). This led us to hypothesize that the plasmid replication begins while the cells are cocci shaped and it segregates when rod shape is attained. Since plasmids use host replication machinery, it is likely that replisome would assemble while the cells are cocci shaped.

### Localization of DnaB-GFP

**Fig 3:**
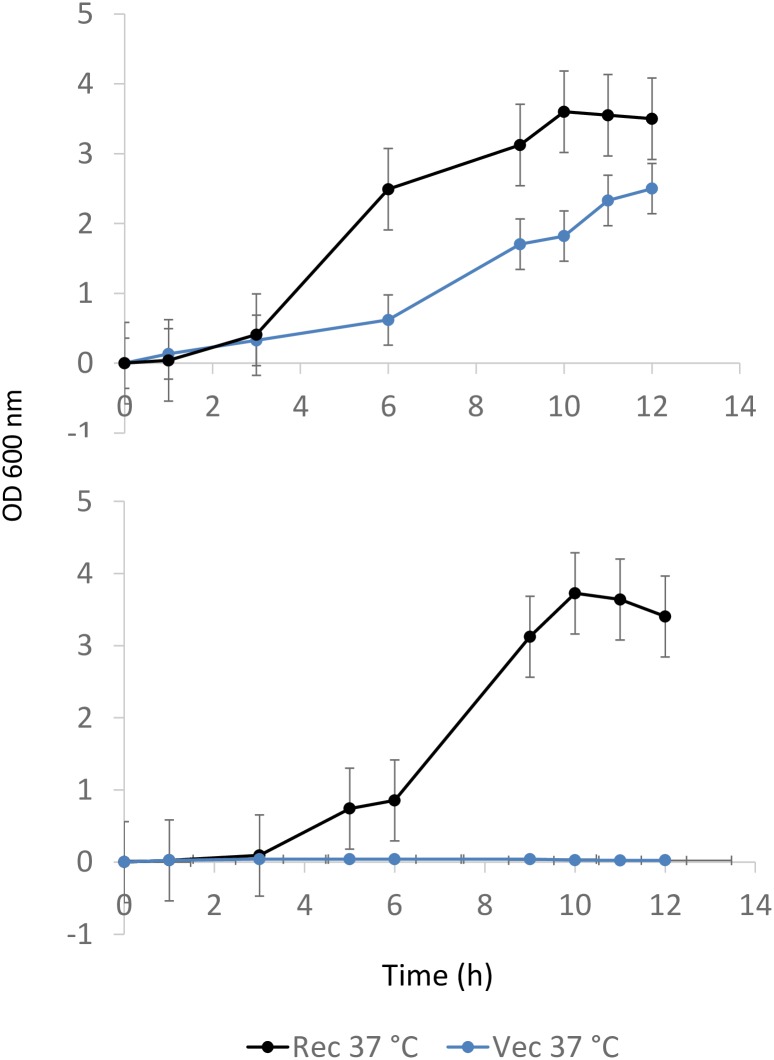
Complementation of phenotype in *E*. *coli* dnaBts achieved by supplying DnaB from *R*. *erythropolis* PR4 fused to GFP via plasmid. The wild type *E*. *coli* could not grow at 37°C whereas the one containing *R*. *erythropolis* dnaB-GFP could grow at 37°C.

**Fig 4:**
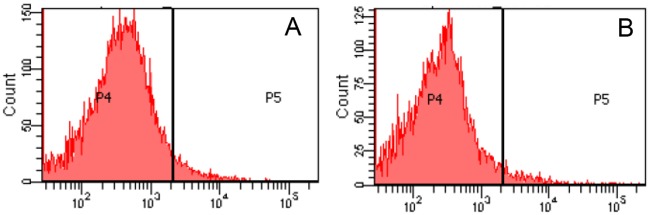
Flow cytometry of wild type *R*. *erythropolis* PR4 (A) and *R*. *erythropolis* PR4 cells containing plasmid which supplies DnaB-GFP (B).

To verify whether replication begins in a cocci cell, we localized the replisomes. For this purpose, DnaB helicase from *R*. *erythropolis* PR4 was supplied in trans via plasmid. A functional fusion of DnaB-GFP was constructed. The functionality of the fusion protein was determined by transforming the plasmid expressing DnaB-GFP in an *E*. *coli* dnaBts strain. *E*. *coli* dnaBts strain can grow at 30°C but not at 37°C. However, *E*. *coli* dnaBts strain transformed with the plasmid expressing DnaB-GFP could grow at 37°C in LB media complementing the phenotype (Fig 3A and 3B). The results suggest that DnaB from *R*. *erythropolis* was functional in *E*. *coli* and also the fusion with GFP did not perturb the activity/functionality of DnaB. Since DnaB-GFP was supplied in trans via plasmid in wild type *R*. *erythropolis* cells, it is likely that DnaB will be present in excess in the cells. In such a situation when replication proteins are in excess, usually there is slight asynchrony. To observe this, replication run off experiment was carried out and the DNA content was compared with the wild type *R*. *erythropolis* cells. Although the run off was not complete, but under identical conditions, it was found that there was only a slight increase in the DNA content of cells containing plasmid expressing DnaB-GFP (Fig 4A and 4B).

**Fig 5:**
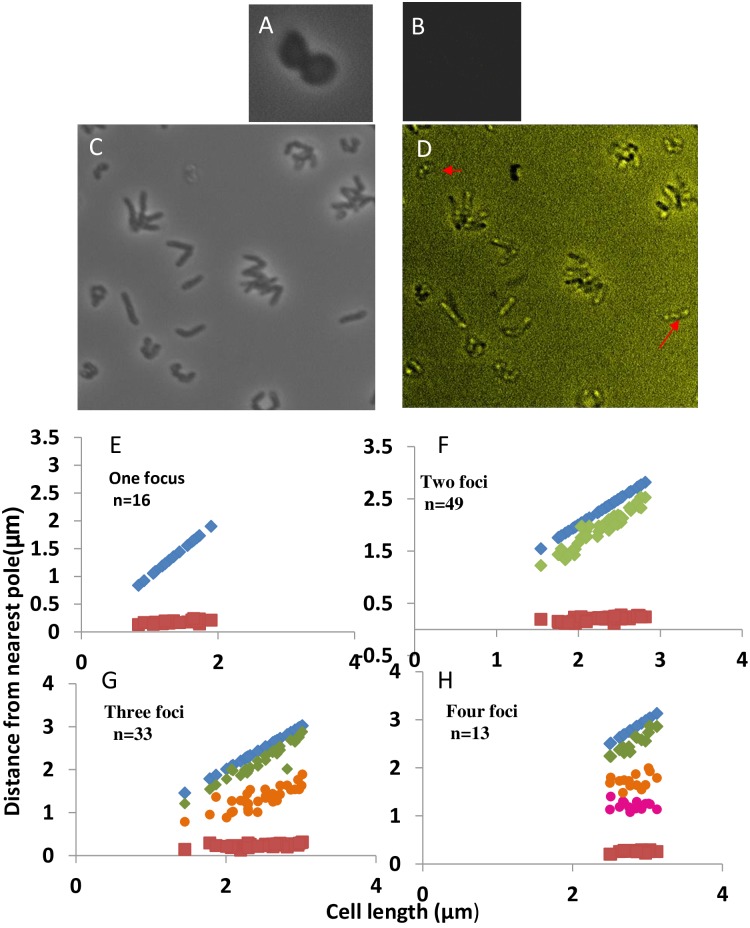
Localization of DnaB-GFP in cocci and rod shaped cells. A) Phase image and B) fluorescence image of cocci cells *of R*. *erythropolis* containing plasmid expressing DnaB-GFP. C) Phase image and D) shows fluorescence in rod shaped cells, Arrow shows one and four foci cells; E-H) Subcellular distribution of DnaB-GFP in *R*. *erythropolis* PR4 grown in LB medium at 30°C.

The cells expressing DnaB-GFP were visualized under the microscope. Surprisingly, none of the cocci cells showed fluorescence foci (Fig 5A and 5B). Cells in their transition from cocci to rod shape displayed fluorescence. The fluorescence for DnaB-GFP was observed near the poles as well as at the cell center (Fig 5C and 5D). Cells displayed one, two, three or four fluorescence foci. The number of cells containing one, two, three and four foci were 16, 49, 33 and 13 respectively (Fig 5E–5H). It is likely that the cell would contain chromosome associated replisome as well as plasmid associated replisome [20]. The number of fluorescence foci observed with plasmid are one to two, however, the number of DnaB-GFP foci are one, two, three or four. Thus the number of foci in the latter case are more perhaps because some of the foci correspond to chromosome associated replisomes.

The smallest cell exhibiting fluorescence was also 1 μm in length. This is almost the same length when two foci cells corresponding to plasmid were observed. Cocci cells displayed diffuse fluorescence or no fluorescence suggesting that cocci cells are non-replicating. Cells which were in their transition from cocci to rod shape however, showed fluorescence of DnaB-GFP further confirming that replication is triggered only when the switch to rod shape is turned on.

### Localization of plasmid pRC4 with respect to nucleoid in filaments

**Fig 6:**
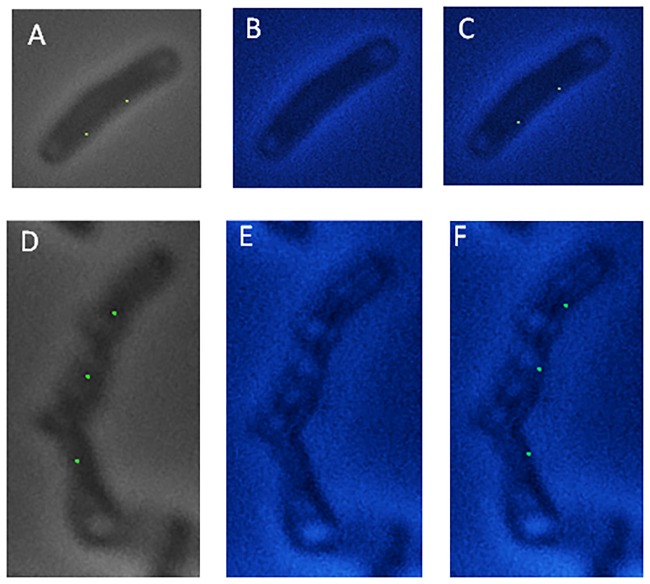
Fluorescence microscopy images showing plasmid pDS3 in cells treated with different concentrations of cephalexin. Nucleoids are stained with DAPI. Top panel: wild type cells showing GFP (A) stained with DAPI (B) and overlay of plasmid and nucleoid (C). Bottom panel: Cells treated with cephalexin 40 μg/ml showing GFP fluorescence (D), nucleoids stained with DAPI (E) and overlay of nucleoids and plasmid (F).

To determine the positioning of plasmids with respect to nucleoid, the cells were stained with DAPI and analyzed. It was found that the fluorescent foci were present in a nucleoid free region (Fig 6A–6C). To determine whether plasmid segregation is independent of cell division, the cells were treated with cephalexin, which is known to prevent cell division. Different concentrations of cephalexin resulted in cells of variable length and branching. At a concentration of 40 μg/ml cephalexin, the cells showed long filaments and branching (data not shown). It was found that plasmid pDS3 was localized in nucleoid free region in long filaments at 40 μg/ml cephalexin (Fig 6D–6F). It was found that the foci were not uniformly distributed in all the cells suggesting that plasmid pRC4 segregation also utilizes different cellular structure, which differ in their sensitivity to cephalexin.

### Segregation of plasmid pRC4 in *R*. *erythropolis*

**Fig 7:**
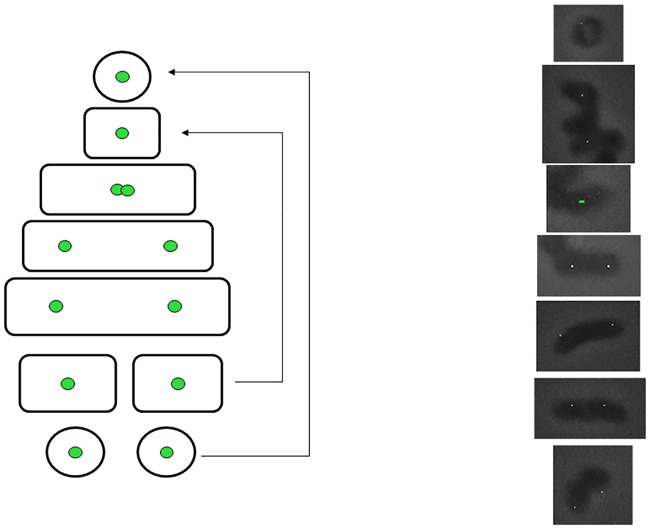
Segregation of plasmid pDS3 with respect to cell cycle. Fluorescence images of representative cells with one or two foci are shown.

The results suggest that the plasmid pRC4 is replicated in the center of the cell and the replicated plasmid rapidly moves to the quarter positions as the cell length increases (Fig 7). The localization of DnaB-GFP suggests that cocci cells are non-replicating and as soon as they become short rods replication begins which is evident from the appearance of DnaB-GFP fluorescence. The mechanism of plasmid segregation at present is unclear. Since the plasmid does not contain partitioning proteins, it is likely that host cytoskeletal structures assist in segregation and thereby its maintenance.

## Discussion

The present work was undertaken to understand the dynamics of plasmid segregation in *R*. *erythropolis*. There are several Rhodococci strains, but *R*. *erythropolis* PR4 is selected, as it is the completely sequenced strain and therefore targeted genome manipulation is possible.

Rhodococci are known to harbor both small and mega plasmids. We initiated studies on localization of small plasmids and selected plasmid pDS3 containing pRC4 replicon for several reasons 1) Plasmid pRC4 has earlier been demonstrated to be stably maintained in *R*. *erythropolis*, 2) Plasmid pRC4 is small, low copy number plasmid (2–4 copies per genome) which replicates via rolling circle replication [21]. There are no reports published on the localization of small plasmids in *Rhodococci*.

There are two methods reported for visualization of plasmid DNA in growing cells, FROS and parS-ParB-GFPsystem. FROS has been demonstrated in case of RK2 [22] and pBET131 [23]. In case of plasmid pBET131 of *Bacillus subtilis*, GFP-LacI tagging resulted in the destabilization of the plasmid [23], thus we decided to use P1 parS-ParB-GFP for tracking plasmid. The system utilizes GFP-Δ30ParB from plasmid P1 which selectively binds to P1 parS and does not interfere with the native segregation machinery of the host chromosome or plasmid [16].

A two-plasmid system was developed where the ParB-GFP was supplied by another plasmid containing pCG1 replicon. Such a method based on the usage of two plasmids has been previously reported for RK2 [22] as well.

In small cells the plasmid was localized in the cell center suggesting that the plasmid pRC4 associate with some protein at the cell center/division septa. Plasmid pRC4 was found to localize at the cell center in small cells and then it goes to the quarter position. The mechanism of plasmid segregation is replicon specific and is usually conserved across bacterial species. In case of plasmid RK2, the localization was found to be conserved in *Vibrio cholerae* and *Pseudomonas aeruginosa* [24]. Localization of plasmids P1, F and RK2 has been shown to be at the cell center in small cells and quarter positions in big cells [24]. High copy number plasmid such as ColE1 on the other hand have been shown to localize at the poles [4].

A functional fusion of DnaB-GFP was constructed. DnaB of *R*. *erythropolis* PR4 is 57% identical to that of *E*. *coli*, yet it could complement the phenotype suggesting that replication proteins are highly conserved across bacteria. Localization of DnaB-GFP showed that rod shaped cells contained fluorescence foci. Small cells contained one fluorescence focus localized at the poles. About 44% cells contained two fluorescence foci localized at the poles. In cells containing three or four fluorescence foci, localization was observed at the poles as well as at the center of the cell. The results suggest that perhaps both plasmid associated as well as chromosome associated replisomes are present. Such plasmid associated replisomes were also observed by Wang et al in case of plasmid pHP13 in *Bacillus subtilis* [20].

Plasmid pRC4 does not encode for any partitioning proteins. Most of the high copy number plasmids have been reported to lack an active partitioning apparatus. They are randomly segregated by diffusing through the cytoplasm and because of the more number of copies; each daughter receives at least one copy and thereby preventing it from being lost. Plasmid pRC4 is a low copy number plasmid [21]. Our findings demonstrate that plasmid pRC4 is stably maintained in *R*. *erythropolis* PR4. It has earlier been demonstrated that the plasmid contains a 0.6 kb BamHI, XhoI fragment which is responsible for stable inheritance [21]. Stable inheritance in small low copy number plasmids has been previously demonstrated for pSC101, pLS11 and pYAN-1 [9].

Plasmid pRC4 has been reported to be present at a copy number of 2–4 per genome [21]. The number of foci however appeared only from 1–2. It is likely that plasmids have aggregated. Such clustering of plasmids has also been observed in case of several plasmids. Even, high copy number plasmids are not randomly located but are present together as clusters [25].

To determine whether plasmids are associated and are present within the nucleoid, DNA was stained with DAPI. It was found that plasmids were localized in a nucleoid free region. Recently, Reyes-Lamothe and coworkers demonstrated that plasmid’s preferred localization depends on its inability to migrate through nucleoid dense regions. Localization to nucleoid-free regions has also been observed for F, R1, RK2 and P1 derivatives lacking functional partition systems [11].

When the cells were treated with cephalexin 40 μg/ml, long filaments were observed and fluorescence foci corresponding to plasmid were distributed suggesting that plasmid segregation is independent of cell division. Plasmid foci, however, were not uniformly distributed in all the filaments. In cephalexin treated cells it was observed that plasmid F was evenly distributed whereas P1 was not. The results suggested that even though P1 and F showed similar localization, they differed with respect to partitioning suggesting that they must be using different cellular factors which differed in their sensitivity to cephalexin [3].

The present work demonstrates for the first time the localization of small plasmids in *Rhodococcus*. Our findings also suggest that the cocci cells in *Rhodococcus* are non-replicating as no fluorescence of DnaB-GFP was observed. The work also established the functionality of parS-ParB-GFP system as a localization tool for following the plasmid and chromosome dynamics in *R*. *erythropolis*.

## Supporting Information

S1 Fig Stability and compatibility of plasmid pDS2 and pDS3. A) Stability of plasmid pDS3; B) Stability of plasmid pDS2 and C) Stability of plasmid pDS2 and pDS3 when both are present together in the same cell.
(TIF)
