# Correction: Localization of Low Copy Number Plasmid pRC4 in Replicating Rod and Non-Replicating Cocci Cells of *Rhodococcus erythropolis* PR4

**DOI:** 10.1371/journal.pone.0171885

**Published:** 2017-02-06

**Authors:** The PLOS ONE Staff

In Fig 5, the cells in image B are not visible. Please see the corrected Fig 5 here. The publisher apologizes for the error.

**Fig 5:**
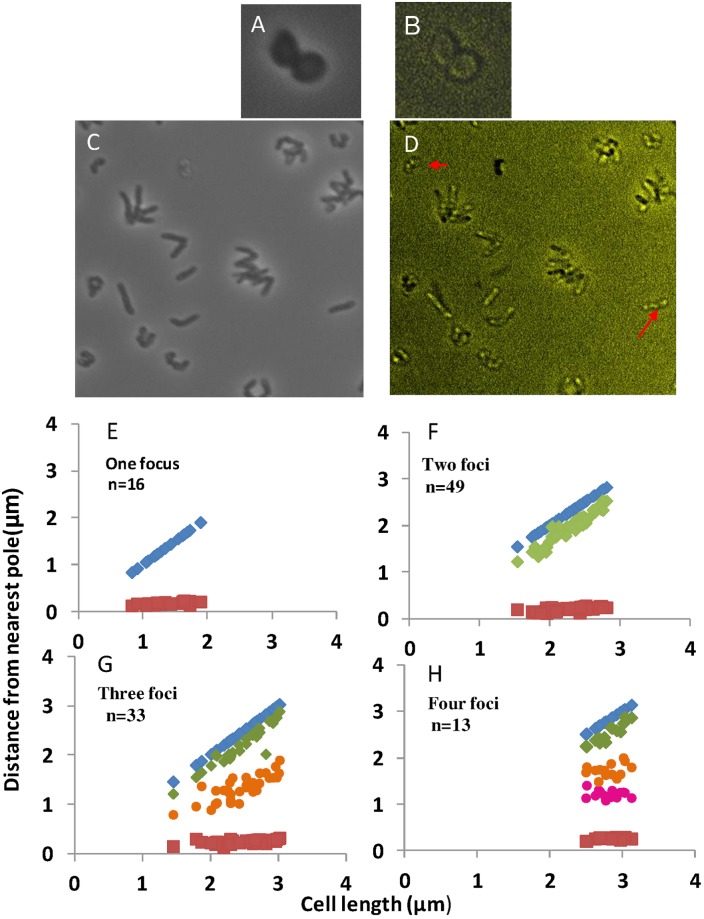
Localization of DnaB-GFP in cocci and rod shaped cells. A) Phase image and B) fluorescence image of cocci cells of *R*. *erythropolis* containing plasmid expressing DnaB-GFP. C) Phase image and D) shows fluorescence in rod shaped cells, Arrow shows one and four foci cells; E-H) Subcellular distribution of DnaB-GFP in *R*. *erythropolis* PR4 grown in LB medium at 30°C.
