# Supplementary figures and images for: Localization of Low Copy Number Plasmid pRC4 in Replicating Rod and Non-Replicating Cocci Cells of Rhodococcus erythropolis PR4
Source: PLoS One. 2016 Dec 9;11(12):e0166491. doi: 10.1371/journal.pone.0166491 (PMC5148583; doi:10.1371/journal.pone.0166491)

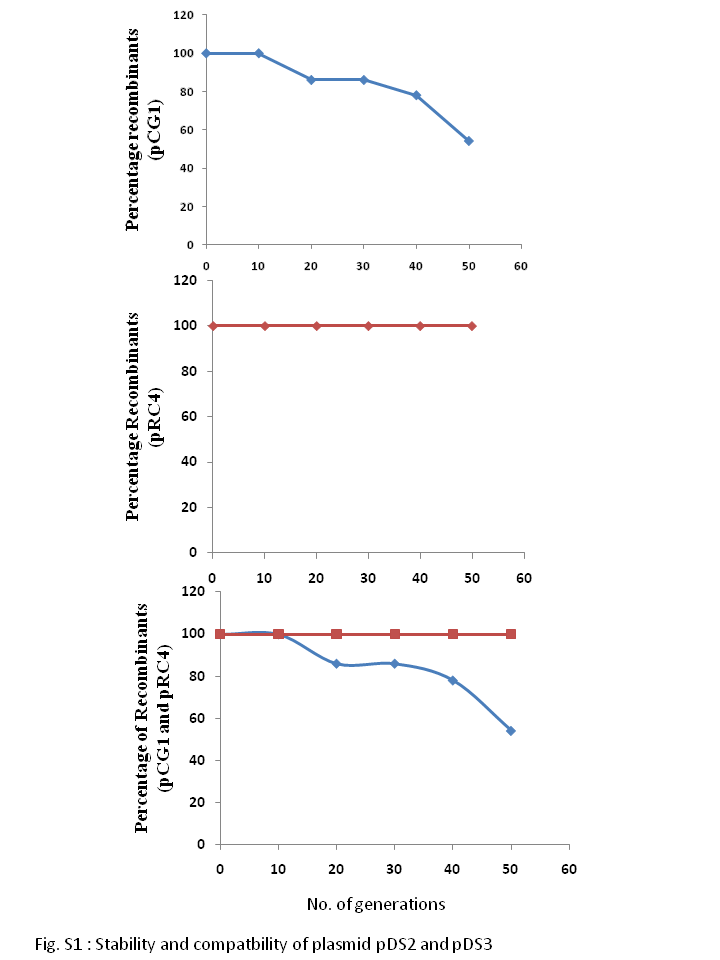

Supplement: S1 Fig — A) Stability of plasmid pDS3; B) Stability of plasmid pDS2 and C) Stability of plasmid pDS2 and pDS3 when both are present together in the same cell. (TIF) [file pone.0166491.s001.tif]
